# Developing and applying a macroinvertebrate‐based multimetric index for urban rivers in the Niger Delta, Nigeria

**DOI:** 10.1002/ece3.5769

**Published:** 2019-10-29

**Authors:** Augustine O. Edegbene, Francis O. Arimoro, Oghenekaro N. Odume

**Affiliations:** ^1^ Unilever Centre for Environmental Water Quality Institute for Water Research Rhodes University Grahamstown South Africa; ^2^ Department of Animal Biology (Applied Hydrobiology Unit) Federal University of Technology Minna Nigeria

**Keywords:** %Chironomidae + Oligochaeta, biomonitoring, Hemiptera abundance, impact categories, MINDU, Niger Delta and water quality

## Abstract

Urban pollution of riverine ecosystem is a serious concern in the Niger Delta region of Nigeria. No biomonitoring tool exists for the routine monitoring of effects of urban pollution on riverine systems within the region. Therefore, the aim of this study was to develop and apply a macroinvertebrate‐based multimetric index for assessing water quality condition of impacted urban river systems in the Niger Delta region of Nigeria. Macroinvertebrate and physicochemical samples were collected from 11 stations in eight river systems. Based on the physicochemical variables, the stations were categorized into three impact categories namely least impacted stations (LIS), moderately impacted stations (MIS) and heavily impacted stations (HIS). Seventy‐seven (77) candidate metrics were tested and only five: Hemiptera abundance, %Coleoptera + Hemiptera, %Chironomidae + Oligochaeta, Evenness index and Logarithm of relative abundance of very large body size (>40–80 mm) were retained and integrated into the final Niger Delta urban multimetric index (MINDU). The validation dataset showed a correspondence of 83.3% between the index result and the physicochemically‐based classification for the LIS and a 75% correspondence for the MIS. A performance of 22.2% was recorded for the HIS. The newly developed MINDU proved useful as a biomonitoring tool in the Niger Delta region of Nigeria and can thus be used by environmental managers and government officials for routine monitoring of rivers and streams subjected to urban pollution.

## Graphical abstract

Macroinvertebrate‐based urban multimetric index was developed for Niger Delta region of Nigeria. The newly developed multimetric index proved useful for assessing ecological health of river systems subjected to urban pollution.

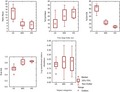

## 1 INTRODUCTION

In sub‐Saharan Africa, due to population increases and industrialization, urbanization is increasing at an alarming rate (Parienté, 2017). While there is a need for rapid urbanization to provide employment for sub‐Saharan African growing population, the unintended consequences of such developments include the pollution and degradation of freshwater ecosystems. The consequences of such pollution include deteriorating water quality, impaired ecological conditions and overall functionality of impacted urban rivers and streams (Edegbene, Arimoro, Odoh, & Ogidiaka, 2015; Edegbene, Elakhame, Arimoro, Osimen, & Odume, 2019; Gieswein, Hering, & Lorens, 2019; Mereta, Boets, Meester, & Goethals, 2013). The Niger Delta, which is home to a range of creeks, rivers and streams, is no exception, as the majority of urban rivers in the region are seriously impacted (Arimoro & Ikomi, 2008). Despite the growing urban pollution in the Niger Delta region, no biomonitoring tool exists for assessing and monitoring the extent of the effects of urban pollution on riverine ecosystems. The development of an appropriate biomonitoring tool can contribute to managing pollution through effectively monitoring and assessing urban pollution effects on riverine biota.

Globally, there is a move toward the combine use of physicochemical and biological monitoring tools for assessing ecological conditions of riverine ecosystems (Arimoro, Odume, Uhunoma, & Edegbene, 2015; Bonada, Prat, Resh, & Statzner, 2006; Ding et al., 2017; Pešić et al., 2019; Shull, Smith, & Selckmann, 2019; Stevenson, Zalack, & Wolin, 2013). It has been acknowledged that physicochemical monitoring alone is inadequate, as results only represent the time and spot from which samples were collected, as well as being very expensive, particularly if a wide range of variables are to be monitored and analyzed (Edegbene et al., 2019; Odume, Muller, Arimoro, & Palmer, 2012). The inadequacies of physicochemical monitoring alone have necessitated the complementary use of biological monitoring (i.e., biomonitoring) tools and approaches (Arimoro, Ikomi, Nwadukwe, Eruotor, & Edegbene, 2014; Bonada et al., 2006; Serra, Graca, Doledec, & Feio, 2017). Biomonitoring tools/approaches widely used include single biotic indices (e.g., South African Scoring System version 5, Dickens & Graham, 2002) functional feeding group (FFG; e.g., Akamagwuna, Mensah, Nnadozie, & Oghenekaro, 2019; Baptista et al., 2013; Lakew & Moog, 2015; Ntislidou, Lazaridou, Tsiaoussi, & Bobori, 2018), multivariate approaches (e.g., Chowdhury, Gallardo, & Aldridge, 2016; Gieswein et al., 2019; Oliveira, Mugnai, Pereira, Souza, & Baptista, 2019), and multimetric indices (e.g., Bonada et al., 2006; Edegbene et al., 2019; Mereta et al., 2013; Monaghan & Soares, 2012). Of these approaches, the multimetric indices have been shown to perform extremely well particularly because they integrate information and data from multiple dimension of aquatic biota and the ecosystem as a whole (Bonada et al., 2006). Multimetric indices have been developed based on aquatic macrophytes (Aguiar, Feio, & Ferreira, 2011; Zervas, Tsiaoussi, & Tsiripidis, 2018); diatoms (Stevenson et al., 2013); phytoplankton (Katsiapi, Moustaka‐Gouni, & Sommer, 2016; Lugoli et al., 2012; Tsiaoussi, Mavromatic, & Kemitzoglou, 2017; Wu, Schmaz, & Fohrer, 2012); macroinvertebrates (Edegbene et al., 2019; Gieswein et al., 2019; Lu, Wu, Xue, Lu, & Batzer, 2019; Ntislidou et al., 2018); and fish (Petriki, Lazaridou, & Bobori, 2017). Macroinvertebrates are particularly useful for index development because they occupy an important position as consumers, can easily be collected, have high diversity, and are differentially sensitive to a gradient of pollution (Bonada et al., 2006; Odume et al., 2012).

While the majority of macroinvertebrate‐based multimetric indices are developed for general water quality (Pešić et al., 2019; Petriki et al., 2017; Stevenson et al., 2013), the intention in this study is to develop a pollution type‐specific multimetric index for assessing urban rivers water quality impairment in Nigeria. The significance of developing an index specific for urban pollution is based on the realization that Nigeria is urbanizing rapidly, and rivers in the Niger Delta region, in particular, suffer from serious urban pollution effects. Therefore, the aim of this study is to develop and apply a macroinvertebrate‐based multimetric index suitable for assessing and monitoring ecological impairments of urban rivers in the Niger Delta region of Nigeria. This study is the first regional macroinvertebrate‐based multimetric index in Nigeria, where studies on biomonitoring methods development are still scanty. The present study thus adds to the few existing studies on macroinvertebrates multimetric indices for biomonitoring of freshwater ecosystems in sub‐Saharan Africa (e.g., Aura, Kimani, Musa, Kundu, & Njiru, 2017; Chirwa & Chilima, 2017; Edegbene et al., 2019; Lakew & Moog, 2015; Mereta et al., 2013; Odume et al., 2012).

## 2 MATERIALS AND METHODS

### 2.1 The study area

The Niger Delta occupies an area of approximately 70,000 km^2^ in the southern tip of Nigeria. The area is characterized by mangrove swamps, wetlands and inland waters (Umoh, 2008). Biodiversity within the region is high (Adekola & Mitchell, 2011). The region supports a wide range of subsistence inland fisheries and wood logging (Zabbey, Erondu, & Hart, 2010). There are two main seasons: the wet and dry season within the Niger Delta (Arimoro et al., 2015; Edegbene & Arimoro, 2012). The wet season is characterized by extensive and intensive rainfall, which begins in April and ends in September. The dry season is characterized by high temperature, usually between 25°C and 35°C. The dry season starts in October and ends in March. The region is known for oil exploration and exploitation. Drainage system in urban cities within the region is poor, and rivers are often impacted by untreated wastewater, storm water return flow, and run‐offs from informal settlements. All of these imply that urban rivers and streams within the regions are being impacted at an alarming rate.

#### 2.1.1 Study river systems

**Figure 1:**
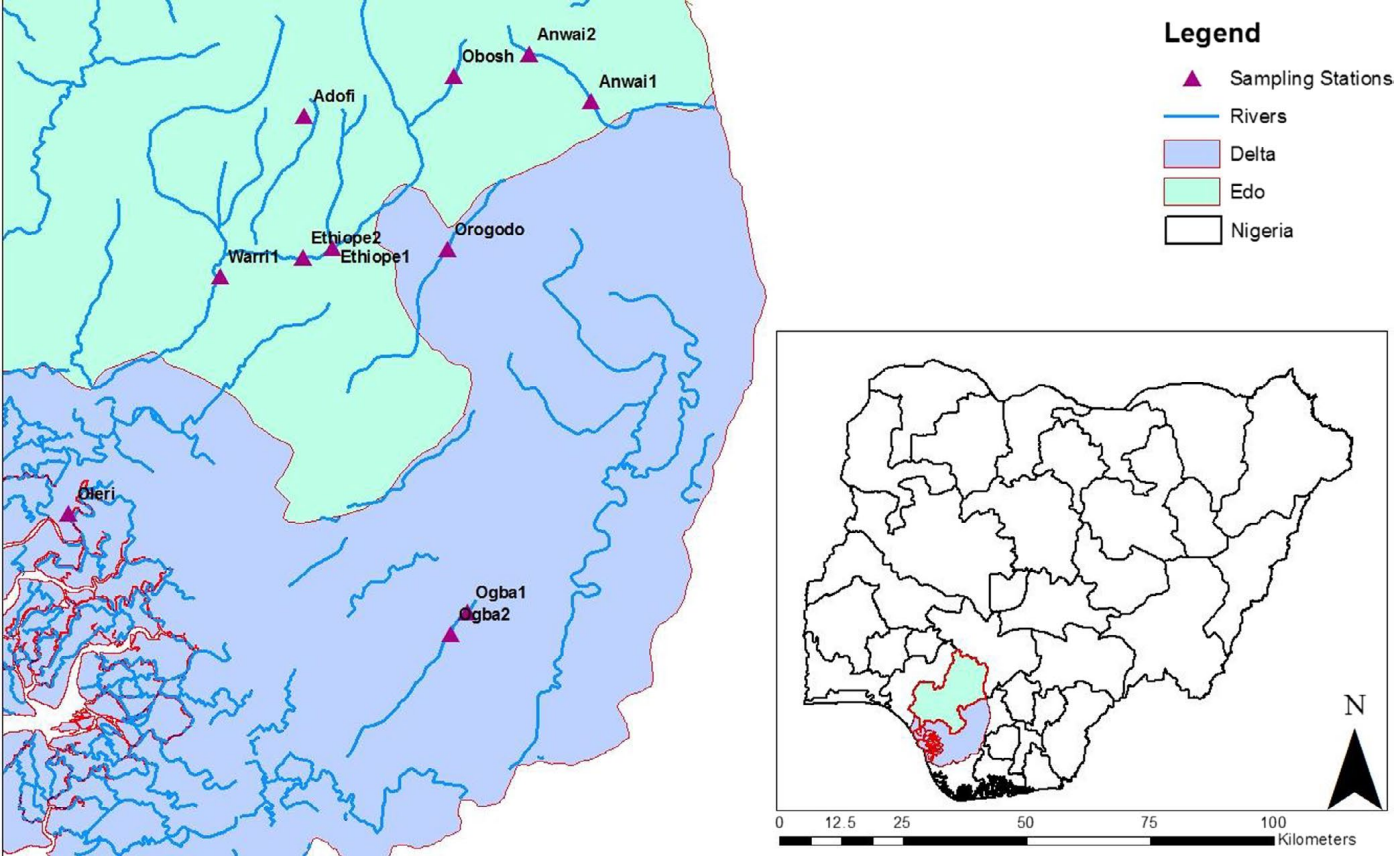
Map of Nigeria showing Delta and Edo States, the sampling stations and rivers within the two states

Eight river systems draining urban landscape in Edo and Delta States within the Niger Delta Region were selected for the study. Samples were collected in 11 stations across the rivers namely Adofi, Anwai (station 1), Anwai (station 2), Ethiope (station 1), Ethiope (station 2), Obosh, Ogba (station 1), Ogba (station 2), Oleri, Orogodo, and Warri Rivers (Figure 1). The rivers are Adofi, Anwai, Ethiope, Obosh, Ogba, Oleri, Orogodo, and Warri Rivers (Figure 1). The rivers were selected on the basis of the degree of urbanization of their landscapes.

### 2.2 Macroinvertebrates and physicochemical sampling

Macroinvertebrates and physicochemical data collected from 2008 to 2012 (five years) were used for the development and validation of the index. Samples were collected monthly in the 11 stations for two seasons, wet season (April–September) and dry season (October–March). Macroinvertebrate data collected from 2008 to 2010 were used for the development of the multimetric index, and those from 2011 to 2012 were used for its validation.

Macroinvertebrates samples were collected using a D‐frame kick‐net (500 µm mesh size; Lazorchak, Klemm, & Peck, 1998). Macroinvertebrates samples were collected at each sampling station for a period of 3 min per biotope. Samples of macroinvertebrate collected from vegetation, sand, silt, mud, and stones were grouped as composite samples and thereafter preserved in 70% alcohol for onward transfer to the laboratory for sorting, identification, and enumeration. Macroinvertebrates were identified to the family level under a stereoscopic microscope at ×10 magnification. Taxonomic guides by Merritt et al. (1996), Day, Harrison, and Moor (2003), and de Moor, Moor, Day, and Moor (2003) were used for the identification.

Physicochemical data were also collected alongside the biological data throughout the sampling period. Physicochemical parameters analyzed for this study were as follows: water temperature, depth, flow velocity, electrical conductivity (EC), pH, dissolved oxygen (DO), five‐day biochemical oxygen demand (BOD_5_), nitrate, and phosphate. A calibrated stick was used in determining the depth of the water in meter. Flow velocity was measured according to Gordon, McMahon, and Finlayson (1994) method. Dissolved oxygen (DO) was measured using dissolved oxygen meter (YSI 55 dissolved meter), while water temperature, pH, and EC were determined using a portable HANNA HI 9913001/1 instrument. Nitrate, phosphate, and BOD_5_ were determined in the laboratory using APHA (1995) methods.

### 2.3 Statistical analyses

#### 2.3.1 Delineation of stations along an urban impact gradient

**Figure A1:**
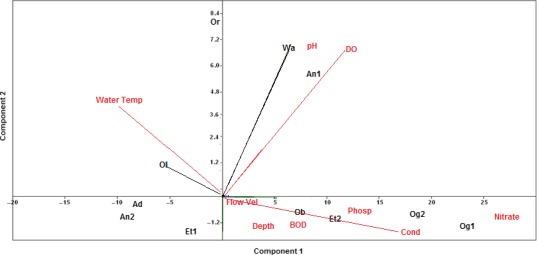
PCA co‐variation showing stations along the impact gradient of physicochemical variables. BOD, five‐day biochemical oxygen demand; Cond, electrical conductivity; DO, dissolved oxygen; Flow Vel, flow velocity; Phosp, phosphate; Water Temp, water temperature

The 11 stations in the eight river sampled were delineated along an urban impact gradient into three impact categories namely least impacted stations (LIS), moderately impacted stations (MIS), and heavily impacted stations (HIS; Table 1). This was achieved by correlating the physicochemical data with the selected river stations using principal component analysis (PCA; Figure A1). Stations strongly correlated with physicochemical indicators of urban pollution such as high nutrients, BOD_5_, and high EC were deemed heavily impacted, and those positively correlated with indicators of good water quality such as high DO were deemed least impacted. The exact categorization was undertaken by extracting the station coordinates on the first axis of the PCA, and then, the interstation distances calculated by subtracting the least scoring station from the highest scoring station. Scores of subsequent stations were then subtracted from the highest scoring station. The interstation distances were converted to percent distances, after which a percentile distribution was used to categorize stations into one of three impact categories: LIS, MIS, and HIS. The percentile distribution for each of the impact categories were 100–90th (LIS), <90th–50th (MIS), and <50th (HIS). A similar method has been used by Murphy, Davy‐Bowker, McFarland, and Ormerod (2013) and Odume, Palmer, Arimoro, and Mensah (2016) to calculate species distances along the first axis of a canonical correspondence ordination plane (CCA). PCA ordination was performed using vegan package version 2.5.4 in R‐statistics (Oksanen et al., 2019).

**Table 1:** Categorization of stations into potential impact categories along the gradient of increasing urban pollution

Major stressor	Rivers/stations Codes	Stations coordinates on PCA axis 1	Interstations distance	% interstations distance	Stations impact category	River stations/impact category codes
Urbanization	Wa	−19.811	42.72	100	1	LIS
	An1	−11.592	34.501	80.76077	1	LIS
	An2	−9.4896	32.3986	75.83942	2	MIS
	Ad	−8.3649	31.2739	73.20669	2	MIS
	Ol	−5.7767	28.6857	67.14817	2	MIS
	Et1	−2.1216	25.0306	58.59223	2	MIS
	Et2	10.287	12.622	29.54588	3	HIS
	Ob	7.0565	15.8525	37.10791	3	HIS
	Og1	22.909	0	0	3	HIS
	Og2	17.97	4.939	11.56133	3	HIS
	Or	−1.0664	23.9754	56.12219	3	HIS

Note

Station impact category: 1 = LIS, 2 = MIS, and 3 = HIS.

Abbreviations: River/stations: Ad, Adofi River; An1, Anwai River station 1; An2, Anwai River station 2; Et1, Ethiope River station 1; Et2, Ethiope River station 2; Ob, Obosh River; Og1, Ogba River station 1; Og2, Ogba River station 2; Ol, Oleri River; Or, Orogodo River; Wa, Warri River. River/stations/impact category codes: HIS, heavily impacted stations; LIS, least impacted stations; MIS, moderately impacted stations.

### 2.4 Metrics selection for multimetric index development

Seventy‐seven (77) candidate metrics were compiled (Table A1), which takes into account various community structure of macroinvertebrates including measures of absolute abundance, composition, richness, diversity, and traits (Baptista et al., 2007; Edegbene et al., 2019; Fierro, Arismendi, Hughes, Valdovinos, & Jara‐Flores, 2018; Mereta et al., 2013; Odume et al., 2012). Trait information was obtained from Krynak and Yates (2018) and Odume, Ntokolo, Akamagwuna, Dallas, and Barber‐James (2018). A fuzzy coding system of 0–3 affinity scores was used to award trait information to macroinvertebrate taxa (Chevenet, Dolédec, & Chessel, 1994). A score of 0 was awarded to a taxon if the taxon has no affinity to the trait attribute, 1 was awarded if the affinity was low, 2 if the affinity was moderate, and 3 if it was high (Chevenet et al., 1994). Measures of abundance was included as part of candidate metrics to be tested in order to represent all component of macroinvertebrate community structures.

### 2.5 Index development

Five steps were followed in developing the index, and these include subjecting all candidate metrics to (a) sensitivity test, (b) seasonality test, (c) redundancy test, (d) integration of selected metrics into the multimetric index, and (e) index validation.

#### 2.5.1 Sensitivity test

Candidate metrics were tested for their potential to discriminate between the LIS from the MIS and HIS. Box plots were used to visualize the metrics. Two levels of discrimination were considered satisfactory. First, a metric was deemed sensitive if there was an overlap between the interquartile ranges (IQRs) of the MIS and HIS, and those of the LIS, but the medians are outside of the interquartile ranges (Edegbene et al., 2019; Odume et al., 2012). Second, a metric was considered sensitivity if the IQR of the LIS do not overlap with those of the MIS and HIS (Edegbene et al., 2019; Odume et al., 2012). Metrics that met all or any of the criterion were selected for further testing.

Selected metrics based on the box plot visualization were further tested for significant differences using the Mann–Whitney (*U*) test. Mann–Whitney (*U*) test was used because Kolmogorov–Smirnov test indicated that metrics were non‐normally distributed. Metrics exhibiting a significant difference between the LIS, and the MIS and HIS at *p* < .05 were retained for further analysis (Barbour et al., 1996). Box plots were done using Statistica version 13.4.14 (TIBCO Software Inc., 2018), and Kolmogorov–Smirnov test of normality and Mann–Whitney tests were computed using Paleontological Statistical Package (PAST; Hammer, Harper, & Ryan, 2001).

#### 2.5.2 Seasonality test

Metrics that were deemed sensitive after confirmation with Mann–Whitney test were further subjected to seasonality test for seasonal stability. Box plots were used to visualize metrics' seasonal stability, and the Kruskal–Wallis test was further used to confirm seasonally stable metric (Baptista et al., 2007). Only metric data from the least impacted stations were used for seasonality test to avoid the confounding effect of pollution on seasonal variation of metrics (Edegbene et al., 2019; Odume et al., 2012).

#### 2.5.3 Redundancy test

Redundant metrics convey the same or similar information (Odume et al., 2012). Spearman's rank correlation coefficient (*r*) was performed on the seasonally stable metrics to explore co‐linearity between the metrics. Metrics with correlation values (Spearman's *r* ≥ .78, *p* < .05) were considered redundant (Edegbene et al., 2019). Non‐redundant metrics were selected for integration to the multimetric index. Where two or more metrics were redundant, only one of such metric was selected for inclusion in the multimetric index (Edegbene et al., 2019).

#### 2.5.4 Integration of the metrics into a multimetric index

Prior to integration, selected metrics were standardized by using the minimum value, lower quartile (25%), mid‐quartile (50%), upper quartile (75%), and maximum value of each metric datasets according to the method described in Baptista et al. (2007). Lower, mid, and upper quartiles were computed with Microsoft Excel, 2010 version. Metrics that were predicted to increase with increasing urban pollution were assigned a score of 5 if the metric value was below the upper quartile (75%) of the LIS, a score of 3 was awarded, if metric value is above the 75%, and a score of 1 is awarded, if the metric value is above the maximum value of the LIS. On the other hand, for metrics that were predicted to decrease with increasing urban pollution, a score of 5 was awarded if metric value of LIS is greater than or equal to lower quartile (25%), a score of 3 was assigned, if the metric value was between the minimum value and <25% of the LIS, while score of 1 is assigned, if the metric value is lower than the minimum value of LIS.

### 2.6 Validation of the multimetric index

A separate macroinvertebrates dataset sampled in 2011 and 2012 was used to validate the developed multimetric index. To test the efficacy of the developed index, the index score was calculated for the station per sampling occasion from the period 2011–2012. The index performance was assessed by calculating the percent correspondence between the index result and the initial station categorization based on the physicochemical variables. The index performance for the LIS was determined by assessing the percent correspondence of LIS falling in the very good–good water quality categories, that of MIS was assessed by assessing the correspondence of MIS falling in the good–fair water quality categories, and that for HIS was assessed by assessing the correspondence of HIS falling in the fair–very poor water quality. Two‐way analysis of variance (ANOVA) was used to test for significance difference between LIS, MIS, HIS, taking space and season as explanatory factors. ANOVA was computed using Paleontological Statistical Package, PAST (Hammer et al., 2001).

### 2.7 Relating the selected metrics to physicochemical variables

Metrics selected for integration into the multimetric index were correlated with physicochemical variables to visualize their distribution an RDA ordination plane. A test of unimodality and linearity using a detrended correspondence analysis (DCA) returned a gradient length of <3 indicating that the dataset were linear (ter Braak, 1995) and thus an RDA was used for the final ordination. A Monte Carlo test at 999 permutations was used to test for the level of significance between the RDA axes (Legendre & Legendre, 2012). The RDA and Monte Carlo test were computed using vegan package within the R programming environment (Oksanen et al., 2019). Co‐linear physicochemical variables (*r* ≥ .80) were removed from the RDA ordination analysis.

## 3 RESULTS

### 3.1 Urban multimetric index

#### 3.1.1 Sensitivity and seasonal stability tests

**Figure 2:**
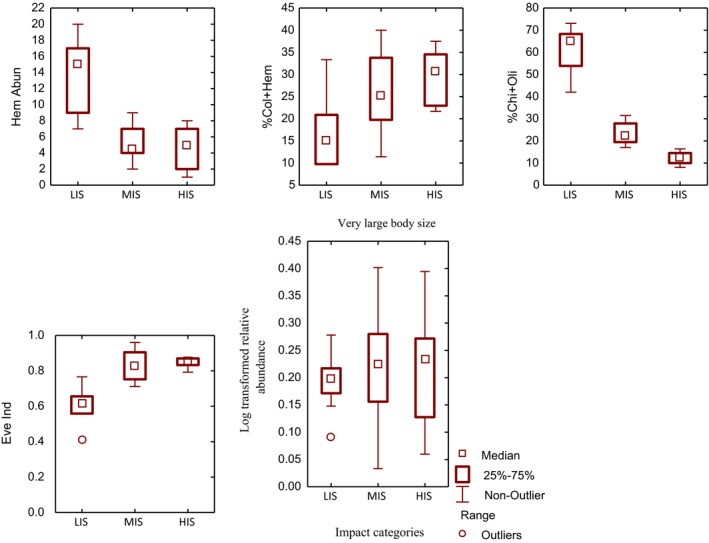
Box plots showing metric discrimination potential of the five metrics integrated into the final multimetric index for urban river assessment in the Niger Delta (MINDU), Nigeria

Of the 77 candidate metrics, only 26 metrics satisfactorily discriminated between the LIS, and the MIS and HIS (Table A2). In all, after subsequent analysis, only five metrics were integrated into the final index, and their discrimination potential are visualized in Figure 2.

**Figure 3:**
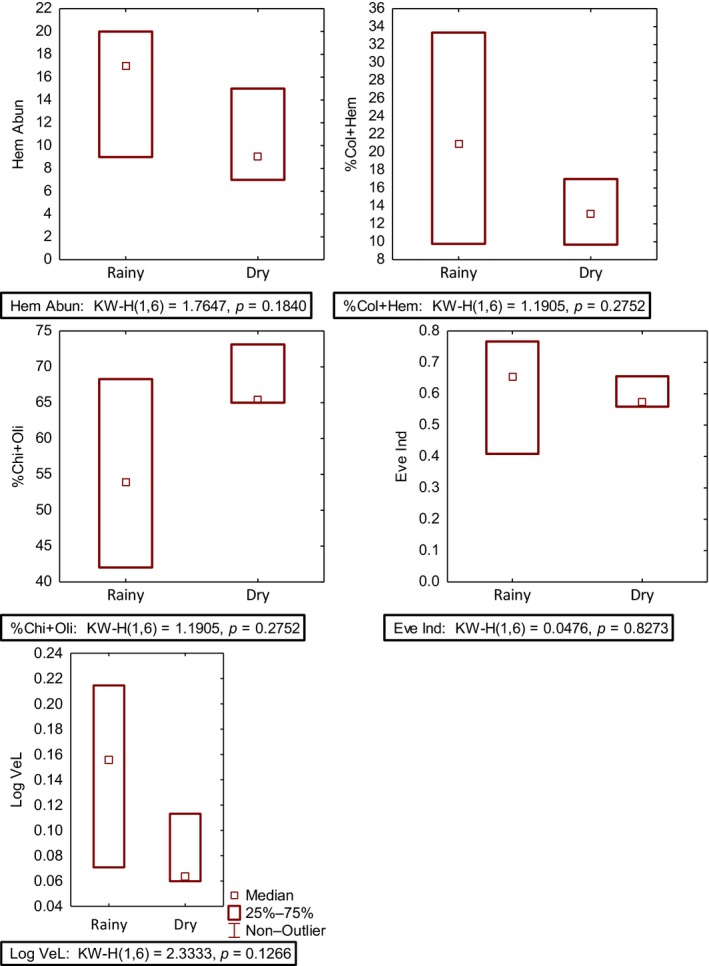
Box plots showing seasonal stability of the five metrics integrated into the final multimetric index development for assessing urban rivers in the Niger Delta (MINDU), Nigeria

Seasonality test indicated that 15 metrics were seasonally stable. The 15 metrics were Chironomidae abundance, Chironomidae + Oligochaeta abundance, Oligochaeta abundance, Hemiptera abundance, Diptera abundance, Mollusca + Diptera abundance, %Chironomidae + Oligochaeta, %Oligochaeta, %Diptera, %Hemiptera, %Coleoptera, %Coleoptera + Hemiptera, %Mollusca + Diptera, Evenness index and logarithm relative abundance of very large body size. Seasonal stability of the five metrics integrated into the multimetric index is shown in Figure 3.

#### 3.1.2 Redundancy test

Apart from the trait measure: very large body size (log VeL), all other sensitive and seasonally stable metrics were found to be redundant with one another (Table A3). However, given that only 15 metrics have been retained thus far and 14 were redundant, and they represent different measures, four of the 14 redundant metrics were retained in addition to log VeL. The four metrics selected in addition to log VeL were Hemiptera abundance, %Coleptera + Hemiptera, %Chironomidae + Oligochaeta and Evenness index (Table A3).

#### 3.1.3 Development of the multimetric index

To develop the multimetric index, the minimum value, lower quartile (25%), mid‐quartile (50%), upper quartile (75%), and maximum value of each metric for the least impacted stations (LIS) metric assemblages values were used as thresholds for calculating the metric scores (Table 2). The multimetric index was computed by summing the scores of the five metrics component, and the index value range (5–25) since five metrics were used (5 × 5 = 25). The index value range then reflect five water quality categories as shown in Table 3.

**Table 2:** Score of metric threshold of the selected metrics for the development of the multimetric index for urban pollution in the Niger Delta, Nigeria

Urban metrics	Statistics					Score		
	Min. value	25%	50%	75%	Max. value	5	3	1
Hem Abun	7	9	12	16.5	20	≥9	7 to <9	<7
%Col + Hem	9.68	10.60	15.05	19.91	33.33	≥10.60	9.68 to <10.60	<9.68
%Chi + Oli	42.03	56.63	65.24	67.60	73.12	<67.60	>67.60 to 73.12	>73.12
Even Ind	0.41	0.56	0.61	0.66	0.77	≥0.56	0.41 to < 0.56	<0.41
Log VeL	0.060	0.065	0.092	0.145	0.21	≥0.065	0.060 to <0.065	<0.060

**Table 3:** Multimetric index score range and associated water quality class for rivers receiving urban pollution in the Niger Delta, Nigeria

Ecological category	Very poor	Poor	Fair	Good	Very good
MINDU score	5–9	10–13	14–17	18–21	22–25
Water quality class	F	E	D	C	B

#### 3.1.4 Validation of the multimetric index

**Figure 4:**
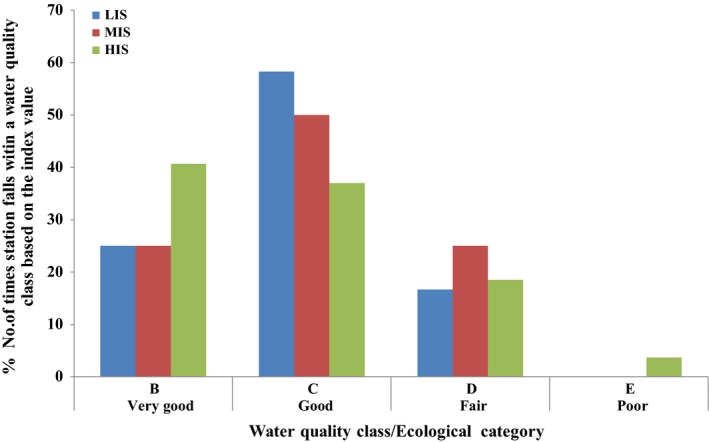
Percent number of times a station category falls within a water quality class based on the MINDU value. HIS, heavily impacted stations; LIS, least impacted stations; MIS, moderately impacted stations. MINDU‐based water quality class: B (very good), C (good), D (fair), E (poor)

The index validation results showed that 25% of the times, stations designated as LIS had very good water quality, and 58.3% of the times, stations designated as LIS had good water quality (Figure 4). Since none of the station could be said to be pristine, the agreement of the classification of the stations based on the physicochemical parameters and the MINDU can be said to be 83.3%, indicating good index performance for the LIS. For the MIS, the index validation results showed that 50% of the times, stations designated as MIS had good water quality and 25% of the times, stations designated as MIS had fair water quality indicating a 75% correspondence between the MINDU results and the physicochemically‐based classification (Figure 4). In terms of the HIS, the validation results indicated that the index performed poorly with only 22.2% (Figure 4) correspondence between the index results and the physicochemically‐based classification, that is, the 18.5% of the times, stations designated as HIS fall in the fair water quality, and 3.7% of the times, stations designated as HIS fall within the poor water quality category. Surprisingly, the index indicated that majority of the times, stations classified as HIS had very good and good water quality compared to the number of times the index indicated that the HIS stations had fair and poor water quality. Nevertheless, the index did perform well for the LIS and MIS stations, as it did not indicate that these stations had poor water quality throughout the sampling period.

**Figure 5:**
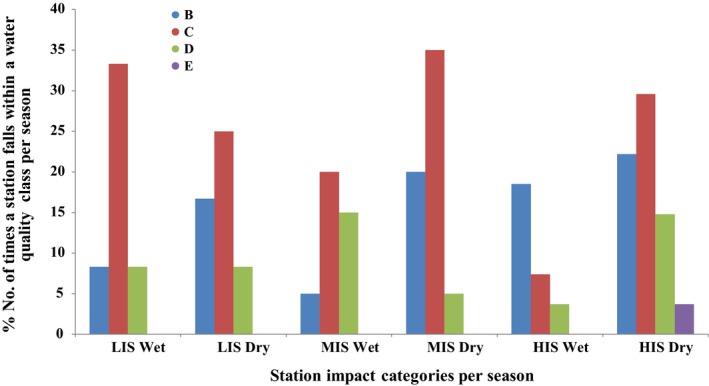
Percent number of times a station category falls within the MINDU‐based water quality class per season (wet and dry). HIS, heavily impacted stations; LIS, least impacted stations; MIS, moderately impacted stations. MINDU‐based water quality class: B (very good), C (good), D (fair), E (poor)

Seasonally, the MINDU results showed that during the wet season, 8.3% of the times, stations designated as LIS had very good water quality and 33.3% of the times, stations designated as LIS had good water quality (Figure 5). The performance of the MINDU was thus 41.6% for the wet season. The dry season performance was 41.7%, with 16.7% and 25% of times, designated stations as LIS had very good and good water quality, respectively (Figure 5). The MINDU performed equally in the wet and dry season for stations designated as LIS.

The seasonal validation results of stations designated as MIS was 20% and 15% of the times good and fair water quality respectively in the wet season while that of the dry season was 35%, and 5% of the times good and fair water quality respectively (Figure 5). It can be said that the MINDU performed more in the dry season (40%) than the wet season (35%) for stations designated as MIS.

Stations designated as HIS in the wet season showed that 3.7% of the times, it had fair water quality (Figure 5). Dry season performance of the MINDU of stations designated as HIS was 18.5% as 14.8% and 3.7% of the times, stations designated as HIS had fair and poor water quality respectively (Figure 5). It can be said that the MINDU performed better in the dry season than in the wet season for stations designated as HIS.

Two ways analysis of variance (ANOVA) indicated no significant differences between LIS, MIS and HIS index value (*p* > .05) while significant difference existed between rainy and dry seasons index values (*p* < .05).

#### 3.1.5 Relating the selected metrics to physicochemical variables

**Figure 6:**
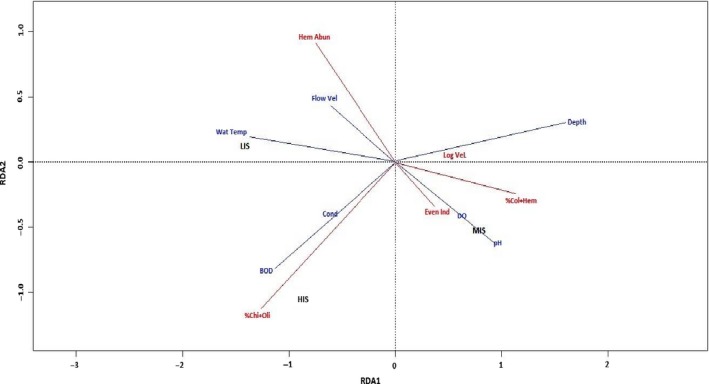
Redundancy ordination plot showing the relationship between macroinvertebrate metrics and physicochemical variables. Metrics: Hem Abun (Hemiptera Abundance), %Col + Hem (%Coleoptera + Hemiptera), %Chi + Oli (%Chironomidae + Oligochaeta), Eve Ind (Evenness Index), Log Vel (logarithm of relative abundance of very large body size). Physicochemical variables: Wat Temp (water temperature), Flow vel (flow velocity), Cond (electrical conductivity), DO (dissolved oxygen), BOD (five‐day biochemical oxygen demand), Depth, and pH. Stations impact categories: LIS (least impacted stations), MIS (moderately impacted stations), and HIS (heavily impacted station)

The first RDA axis explained 86.98% of the ordination plot, while the second axis explained 13.02%. The Eigen value of the first axis was higher, 6.409 compared to the 0.40918 Eigen value of the second axis. There was no significant difference in the two RDA axes correlation with metrics and the physicochemical variables (*p* > .05) as revealed by the Monte Carlo test at 999 permutation. Dissolved oxygen strongly correlated with Evenness index and % Coleoptera + Hemiptera (Figure 6). Logarithm of relative abundance of very large body size was positioned at the centre of the RDA triplot and was correlated with depth. Five‐day biochemical oxygen demand and EC were strongly correlated to % Chironomidae + Oligochaeta at the HIS. Hemiptera abundance was correlated to water temperature and flow velocity at LIS (Figure 6).

## 4 DISCUSSION

A total of 77 macroinvertebrates candidate metrics were tested of which only five representing trait measure, composition, diversity, and abundance were retained and integrated into the final MINDU. Of all the candidate metrics in the various measures considered for integration into the MINDU, 26 metrics were discriminatory and confirmed sensitive. Most of the sensitive metrics were in the abundance and composition measures. The abundance and composition metrics are widely recognized as being sensitive to pollution and therefore often integrated into multimetric indices (Baptista et al., 2013; Gieswein et al., 2019; Huang et al., 2015; Lu et al., 2019; Melo, Stenert, Dalzochio, & Maltchik, 2015).

The diversity measures, Margalef's index, Shannon‐weiner, Simpson diversity, and Evenness index were all discriminatory of the MIS and HIS from the LIS but only Evenness index was confirmed sensitive. Similar studies elsewhere have reported most diversity measures to have high discriminatory potentials (Edegbene et al., 2019; Ntislidou et al., 2018). Edegbene et al. (2019) integrated two diversity measures namely Margalef index and Shannon diversity index into the Chanchaga multimetric index (MMIchanchaga) developed for a river in northern Nigeria. This attest to the fact that diversity measures are useful biomonitoring tools. Other studies have also integrated Margalef index (Mereta et al., 2013) and Shannon diversity (Aura et al., 2017) into macroinvertebrate‐based multimetric indices. In the present study, Evenness index was the only diversity measure integrated into the final index because the remaining measures were found to be either seasonally unstable or redundant. Zamora‐Muniz, Sainz‐Cantero, Sanchez‐Ortega, and Alba‐Tercedor (1995) have cautioned against the use of metrics that are seasonally unstable because of the difficulty of disentangling variation occasioned by natural seasonal dynamics from those occasioned by anthropogenic activities.

One of the five metrics integrated into the final MINDU was trait measure, that is, very large body size (>40–80 mm). Organisms with body size ranging between >40 and 80 mm have proved highly sensitively to urban pollution and was non‐redundant with the rest of the taxonomic metrics. Abundances of very large‐bodied macroinvertebrates have been hypothesized to decrease in response to environmental stress because they are often associated with long reproductive cycle and fewer offspring per reductive event compared to small bodied individuals, which often reproduce rapidly (Castro, Dolédec, & Callisto, 2018; Serra et al., 2017; Townsend & Hildrew, 1994). Studies testing metrics for integration into multimetric indices have often ended up with one or two trait‐based metrics in the final indices, indicating that the present study, which found only a single trait to be highly sensitive and non‐redundant was in accordance with most other studies (e.g., Baptista et al., 2007; Fierro et al., 2018; Gieswein et al., 2019; Ntislidou et al., 2018). The inclusion of the trait‐based metric into the final MINDU is particularly useful because while taxonomic metrics relate to structural measure, traits relate to the functional aspects of the biota (Desrosiers et al., 2019; Ding et al., 2017; Monaghan & Soares, 2012).

The five candidate metrics integrated into the MINDU are sparsely reported as metrics for development of multimetric indices except the %Hemiptera + Coleoptera and Hemiptera abundance (Aura et al., 2017; Edegbene et al., 2019). This informs the selection of %Coleoptera + Hemiptera and Hemiptera abundance for integration into the MINDU even when they were redundant. Furthermore, two metrics from the composition measures were retained, though redundant. The %Coleoptera + Hemiptera reflect moderately tolerant macroinvertebrates taxa while %Chironomidae + Oligochaeta reflect taxa that are tolerant of pollution. Mereta et al. (2013) has also selected final metrics based on their degree of sensitivity to water quality impairment.

The validation of the performance of the developed MINDU with separate datasets revealed that the index performed better for LIS and MIS compared with the HIS. The relatively good performance of the index for the LIS and MIS stations indicates that using the index may not lead to under or over protection, whereas the poor performance of the index for the HIS could be that pollution at these stations are seasonally mediated such that macroinvertebrate recovery and recolonization are rapid, reducing the cumulative effects of pollution. Even though seasonal stability was tested for during the selection of the metrics, it appears that a “flushing effect” aggravated the effects of urban pollution during the wet season. During the wet season, water quality at the HIS was generally poor, compared with the dry season. It is postulated that increased urban storm water run‐off, as well as run‐off from settlements, carrying pollutants may have led to the poor water quality during the wet season at the HIS. Similar findings have been reported by Speak, Rothwell, Lindley, and Smith (2013) that increased urban run‐off due to increased precipitation led to increased pollution of riverine ecosystems. Water quality at the HIS seems to recover during the dry season and thus mediating the overall performance of the developed index. The implication therefore is that monitoring need to be structured to take account of seasonality, and data interpreted taking into account the season‐mediating effects of urban pollution.

The developed MINDU performed better in the dry season than in the wet season except for the LIS. The reason for the better performance of the index during the dry season could be attributed to reduced urban run‐off during the season. Urban run‐off is one of the major factors influencing water quality of rivers in the Niger Delta. In addition, heavy rains have impact on water quality because debris and other pollutants are carried into urban river systems during down pour. In contrast to the findings in the present study, is the work of Edegbene et al. (2019) found that a similar index developed for a river in north central Nigeria did not exhibit much seasonal variation in terms of performance. This may not be unconnected to longer wet seasons in the Niger Delta region compared to the North central region of Nigeria were Edegbene et al. (2019) reported contrary findings.

## 5 CONCLUSION

In the present study, a Niger Delta urban multimetric index (MINDU) was developed for monitoring urban pollution effects in the Niger Delta. Five metrics representing abundance, composition, diversity, and trait measures were integrated into the final index. For the LIS stations, the metric performed very well, recording a 83.3% correspondence with physicochemically‐based station classification. For the MIS, 75% correspondence between the index results and physicochemically‐based classification was recorded, while for the HIS, only 22.2% correspondence was recorded. The newly developed MINDU proved effective as a biomonitoring tool for monitoring river health in the Niger Delta region of Nigeria and can thus be used by environmental managers and government officials for routine monitoring of rivers and streams subjected to urban pollution.

## CONFLICT OF INTEREST

None declared.

## AUTHOR CONTRIBUTIONS

Augustine Ovie Edegbene, Oghenekaro Nelson Odume, and Francis Ofurum Arimoro design the project. Augustine Ovie Edegbene and Francis Ofurum Arimoro collected the data. Augustine Ovie Edegbene analyzed the data. Augustine Ovie Edegbene and Oghenekaro Nelson Odume interpreted the results. Drafting of the article manuscript was done by Augustine Ovie Edegbene and Oghenekaro Nelson Odume. All authors approved the final manuscript.

## Data Availability

Our datasets have been deposited with Dryad, and the DOI accession number is https://doi.org/10.5061/dryad.98sf7m0dq.

## APPENDIX A

**Table A1:** Selected candidate metrics and their predicted response to urban pollution

S/N	Candidate metrics	Metric codes	Predicted response to urban pollution
Abundance measures (absolute number of individuals in macroinvertebrate groups)			
1	Ephemeroptera Plecoptera and Trichoptera abundance	EPT Abun	Decrease
2	Ephemeroptera family abundance	Eph Abun	Decrease
3	Trichoptera family abundance	Tri Abun	Decrease
4	Ephemeroptera Trichoptera Odonata and Coleoptera abundance	ETOC Abun	Decrease
5	Chironomidae abundance	Chi Abun	Increase
6	Chironomidae + Oligochaeta abundance	Chi + Oli Abun	Increase
7	Oligochaeta family abundance	Oli Abun	Increase
8	Diptera family abundance	Dip Abun	Increase
9	Mollusca + Diptera family abundance	Mol + Dip Abun	Increase
10	Decapoda family abundance	Dec Abun	Decrease
11	Mollusca family abundance	Mol Abun	Increase
12	Mollusca + Decapoda family abundance	Mol + Dec Abun	Variable
13	Coleoptera family abundance	Col Abun	Decrease
14	Odonata family abundance	Odo Abun	Decrease
15	Hemiptera family abundance	Hem Abun	Decrease
16	Coleoptera + Hemiptera abundance	Col + Hem Abun	Decrease
17	Ephemeroptera Plecoptera and Trichoptera family/Chironomidae abundance	EPT/Chi Abun	Decrease
18	Ephemeroptera Trichoptera Odonata and Coleoptera family/Chironomidae abundance	ETOC/Chi Abun	Decrease
19	Ephemeroptera Trichoptera Odonata and Coleoptera family/Diptera abundance	ETOC/Dip Abun	Decrease
20	Chironomidae/Diptera family abundance	Chi/Dip Abun	Increase
Composition measures (relative abundance of individual macroinvertebrates in the entire sample)			
21	%Ephemeroptera, Plecoptera, and Trichoptera	%EPT	Decrease
22	%Ephemeroptera	%Eph	Decrease
23	%Ephemeroptera, Trichoptera, Odonata, and Coleoptera	%ETOC	Decrease
24	%Trichoptera	%Tri	Decrease
25	%Chironomidae	%Chi	Increase
26	%Chironomidae + Oligochaeta	%Chi + Oli	Increase
27	%Oligochaeta	%Oli	Increase
28	%Diptera	%Dip	Increase
29	%Decapoda	%Dec	Decrease
30	%Mollusca	%Mol	Increase
31	%Mollusca + Decapoda	%Mol + Dec	Variable
32	%Odonata	%Odo	Decrease
33	%Hemiptera	%Hem	Decrease
34	%Coleoptera	%Col	Decrease
35	%Coleoptera + Hemiptera	%Col + Hem	Decrease
36	%Mollusca + Diptera	%Mol + Dip	Increase
Richness measures (absolute number of taxa of macroinvertebrate group)			
37	Ephemeroptera, Plecoptera, and Trichoptera richness	EPT Rich	Decrease
38	Ephemeroptera richness	Eph Rich	Decrease
39	Trichoptera richness	Tri Rich	Decrease
40	Diptera richness	Dip Rich	Increase
41	Ephemeroptera, Trichoptera, Odonata, and Coleoptera richness	ETOC Rich	Decrease
42	Chironomidae richness	Chi Rich	Increase
43	Chironomidae + Oligochaeta richness	Chi + Oli Rich	Increase
44	Mollusca richness	Mol Rich	Increase
45	Coleoptera + Hemiptera richness	Col + Hem Rich	Decrease
46	Coleoptera richness	Col Rich	Decrease
47	Hemiptera richness	Hem Rich	Decrease
48	Odonata richness	Odo Rich	Decrease
49	Oligochaeta richness	Oli Rich	Increase
50	Decapoda richness	Dec Rich	Decrease
Diversity measures			
51	Simpson diversity (1‐D) (weighted toward the abundance of commonest taxa (Edegbene et al., 2019; Ogbeibu, 2005)	Sim Div	Decrease
52	Evenness index (e^H/S) (evenness of taxa within sample (Clarke & Warwick, 1994)	Eve Ind	Decrease
53	Margalef index (taxa diversity index) (account for both number of taxa and individuals and is independent of sample size (Ogbeibu, 2005))	Mar Ind	Decrease
54	Shannon‐Weiner diversity index (H) (information statistics index taking account of contribution of individual taxa to the diversity while assigning greater weight to dominant taxa (Ogbeibu, 2005))	Sha Ind	Decrease
Traits attributes/ ecological preferences			
Body armoring			
55	Logarithm of the relative abundance of hard shelled individuals	Log HaS	Decrease
56	Logarithm of the relative abundance of individuals with soft and exposed body	Log SoE	Increase
57	Logarithm of the relative abundance of cased/tubed individuals	Log CaT	Increase
Voltinism (no. of generation per year)			
58	Logarithm of the relative abundance of individual completing their life cycle in 1 year (univoltine)	Log Uni	Decrease
59	Logarithm of relative abundance of individual completing their life cycle in 2 years (bivoltine)	Log Biv	Increase
Attachment mechanism			
60	Logarithm of relative abundance of Free‐living	Log FrL	Increase
61	Logarithm of the relative abundance of individuals with features for permanent attachment	Log PeA	Decrease
Mobility			
62	Logarithm of the relative abundance of Crawlers	Log Cra	Decrease
63	Logarithm of relative abundance of Sprawler	Log Spr	Increase
64	Logarithm of relative abundance of Skater	Log Ska	Increase
65	Logarithm of relative abundance of Burrower	Log Bur	Increase
Response to oxygen depletion			
66	Logarithm of relative abundance of individuals showing moderate sensitivity oxygen depletion	Log MoS	Decrease
67	Logarithm of relative abundance of highly tolerant oxygen depletion	Log HiT	Increase
Body sizes/shape			
68	Logarithm of the relative abundance of individuals with very large body sizes (>40–80 mm)	Log VeL	Decrease
69	Logarithm of relative abundance of small, >5–10 mm	Log Sma	Increase
70	Logarithm of relative abundance of cylindrical/tubular body shaped individuals	Log CyT	Increase
Respiration			
71	Logarithm of the relative abundance of individuals using tegument for respiration	Log Teg	Increase
Turbidity preferences			
72	Logarithm of the relative abundance of individuals showing preference for silty/turbid waters	Log SiT	Increase
73	Logarithm of relative abundance of individuals showing no preference for turbid water	Log NoT	Increase
Food preference/feeding habit			
74	Logarithm of relative abundance of individuals feeding on fine particulate organic matter (FPOM)	Log DeF	Increase
75	Logarithm of relative abundance of filter feeders	Log FiF	Increase
Aquatic stages			
76	Logarithm of relative abundance of individuals having larval aquatic stage	Log Lav	Increase
77	Logarithm of relative abundance of individual having pupal aquatic stage	Log Pup	Increase

**Table A2:** Sensitive metrics selection for urban dominated rivers of the Niger Delta, Nigeria, as revealed Mann–Whitney (*U*) test

Discriminatory metrics	Mann–Whitney test (*U*‐test)	*p*‐value	Sensitivity confirmed
Abundance measures			
EPT Abun	70.5	0.9539	No
Eph	62.0	0.582	No
ETOC Abun	72.0	0.02889	Yes
Chi Abun	3.0	7.36E−05	Yes
Chi + Oli Abun	6.0	0.0001517	Yes
Oli Abun	15.0	0.0009811	Yes
Dip Abun	2.0	5.722E−05	Yes
Mol + Dip Abun	2.0	5.722E−05	Yes
Dec Abun	34.5	0.02636	Yes
Col Abun	70.5	0.9539	No
Odo Abun	69.5	0.9077	No
Hem Abun	22.5	0.004344	Yes
EPT/Chi Abun	25.5	0.007858	Yes
ETOC/Chi Abun	27.0	0.01019	Yes
ETOC/Dip Abun	19.0	0.002437	Yes
Chi/Dip Abun	32.5	0.02367	Yes
Composition measures			
%EPT	43.0	0.09988	No
%Eph	52.0	0.2601	No
%ETOC	44.0	0.1123	No
%Chi	33.0	0.0262	Yes
%Chi + Oli	29.0	0.01414	Yes
%Oli	4.0	8.232E−05	Yes
%Dip	25.0	0.00726	Yes
%Dec	25.0	0.004756	Yes
%Odo	52.0	0.2598	No
%Hem	8.0	0.0002155	Yes
%Col	24.0	0.006099	Yes
%Col + Hem	26.0	0.008616	Yes
%Mol + Dip	24.0	0.006099	Yes
Richness measures			
EPT Rich	64.5	0.684	No
Tri Rich	41.0	0.06599	No
Dip Rich	55.5	0.3417	No
ETOC Rich	64.0	0.6843	No
Chi Rich	43.0	0.08061	No
Chi + Oli Rich	12.0	0.0004453	Yes
Col + Hem Rich	67.0	0.7917	No
Col Rich	60.5	0.5202	No
Odo Rich	63.0	0.6173	No
Oli Rich	6.5	0.0001184	Yes
Dec Rich	36	0.01805	Yes
Diversity measures			
Sim Div	62.5	0.6033	No
Sha Div	71.0	0.977	No
Eve Div	1.0	4.695E−05	Yes
Mar Div	68	0.8399	No
Traits attributes measures			
Log HaS	10.0	0.931	No
Log VeL	70.0	0.0002652	Yes

Note

A metric sensitivity was confirmed if significant at *p* < .05.

**Table A3:** Spearman's rank correlation of macroinvertebrate metrics showing co‐linearity between metrics (*r* ≥ .78, *p* < .05)

	Chi Abun	Chi + Oli Abun	Oli Abun	Hem Abun	Dip Abun	Mol + Dip Abun	%Chi + Oli	%Oli	%Dip	%Hem	%Col	%Col + Hem	%Mol + Dip	Eve Ind	LogVeL
Chi Abun	0	2.9E−15	6.0E−06	0.0077	1.9E−16	1.1E−15	8.7E−05	2.2E−05	8.1E−05	0.00014	0.00054	0.013	3.2E−05	0.00023	0.23
Chi + Oli Abun	**0.97**	0	3.1E−08	0.0017	1.8E−15	9.0E−19	3.5E−05	5.3E−06	6.5E−05	2.0E−05	0.00011	0.0144	2.3E−05	0.00015	0.19
Oli Abun	**0.78**	**0.87**	0	0.00017	1.2E−06	3.0E−07	0.0010	5.2E−06	0.0022	1.3E−06	13E−05	0.014	0.0014	8.6E−06	0.081
Hem Abun	0.53	0.61	0.69	0	0.0028	0.030	0.055	0.00078	0.023	5.02E−06	0.0011	0.041	0.020	0.00016	0.66
Dip Abun	**0.98**	**0.97**	**0.82**	0.58	0	6.4E−19	0.00043	7.9E−06	0.00040	1.4E−05	0.0015	0.034	0.00021	9.8E−05	0.35
Mol + Dip Abun	**0.97**	**0.98**	**0.84**	0.58	**0.99**	0	7.6E−05	1.3E−06	0.00011	2.7E−05	0.00052	0.016	4.4E−05	6.2E−05	0.32
%Chi + Oli	0.71	0.74	0.63	0.40	0.66	0.72	0	0.0018	7.4E−14	0.039	0.0010	0.031	2.4E−15	0.0057	0.013
%Oli	0.75	**0.79**	**0.79**	0.64	**0.78**	**0.81**	0.60	0	0.0039	8.0E−05	6.0E−06	6.7E−05	0.0022	7.1E−08	0.36
%Dip	0.72	0.72	0.59	0.46	0.66	0.71	**0.96**	0.57	0	0.025	0.0041	0.034	1.4E−22	0.0045	0.022
%Hem	0.70	0.76	**0.81**	**0.79**	0.76	0.75	0.42	0.72	0.46	0	0.001	0.14	0.019	5.7E−05	0.54
%Col	−0.65	−0.71	−0.77	−0.62	−0.61	−0.65	−0.63	**−0.78**	−0.56	−0.63	0	3.4E−06	0.0021	3.9E−05	0.076
%Col + Hem	−0.50	−0.49	−0.49	−0.42	−0.43	−0.48	−0.44	−0.72	−0.43	−0.31	**0.80**	0	0.033	7.1E−05	0.15
%Mol + Dip	0.74	0.75	0.61	0.47	0.69	0.73	**0.97**	0.59	**0.99**	0.48	−0.60	−0.43	0	0.0047	0.024
Eve Ind	−0.68	−0.69	−0.78	−0.69	−0.71	−0.72	−0.55	**−0.86**	−0.56	−0.73	0.74	0.72	−0.56	0	0.47
LogVeL	−0.26	−0.28	−0.36	−0.093	−0.20	−0.21	−0.50	−0.19	−0.47	−0.13	0.37	0.30	−0.46	0.15	0

Note

Bold values were significant at *p* < .05.

Chi Abun (Chironomidae abundance), Chi + Oli Abun (Chironomidae + Oligochaeta abundance), Oli Abun (Oligochaeta abundance), Hem Abun (Hemiptera abundance), Dip Abun (Diptera abundance), Mol + Dip (Mollusca + Diptera abundance), %Chi + Oli (percentage Chironomidae + Oligochaeta), %Oli (percentage Oligochaeta), %Dip (percentage Diptera), %Hem (percentage Hemiptera), %Col (percentage Coleoptera), %Col + Hem (percentage Coleoptera + Hemiptera), %Mol + Dip (percentage Mollusca + Diptera), Eve Ind (Evenness index), LogVeL (logarithm of relative abundance of very large body size [>40–80]).
